# Study on the impact of exogenously applied methyl jasmonate concentrations on *Solanum lycopersicum* under low temperature stress

**DOI:** 10.1186/s12870-023-04449-8

**Published:** 2023-09-19

**Authors:** Nadia Gul, Khalid Z Masoodi, Salika Ramazan, Javid I Mir, Saima Aslam

**Affiliations:** 1 Department of Biotechnology, School of Biosciences and Biotechnology, Baba Ghulam Shah Badshah University, Rajouri, 185234 India; 2 Transcriptomics Laboratory (K-Lab), Division of Plant Biotechnology, Sher-eKashmir University of Agricultural Sciences and Technology of Kashmir, Shalimar, 190025 India; 3 Department of Botany, University of Kashmir, Srinagar, 190006 India; 4 Department of Plant Biotechnology, Central Institute of Temperate Horticulture (CITH), Rangreth, Srinagar, 190007 J&K India

**Keywords:** Jasmonic acid, *Solanum lycopersicum*, Methyl jasmonate, Low temperature stress

## Abstract

**Background:**

To decipher the capability of Methyl Jasmonate (MeJA) in resisting cold stress in *Solanum lycopersicum* assessment regarding various physiological parameters in response to diverse doses of MeJA was done. Low temperature (LT) were given to the plants with MeJA (J1C, J2C, J3C) or without MeJA (LT) application. MeJA in the form of foliar spray was given before stress, during stress and after stress. Three concentrations of MeJA were used under normal and LT stress conditions that includes of J1 (0.5 µM), J2 (10 µM), and J3 (15 µM).

**Results:**

Oxidative stress, growth characteristics, stress tolerance parameters, antioxidant response and photosynthetic parameters were investigated. In our current study we observed that oxidative stress markers declined by MeJA supplementation under cold stress conditions. MeJA boosted antioxidant enzyme activity along with photosynthetic parameters. The best concentration of MeJA was J2 based on results obtained. This is the first study related to MeJA best dose screening in *Solanum lycopersicum* under LT stress conditions.

**Conclusion:**

The LT stress in the *Solanum lycopersicum* plant was reduced by MeJA. The adverse consequences of LT stress can be significantly attenuated by the J2 concentration of MeJA. So, the optimal concentration of MeJA supplied exogenously to LT stressed *Solanum lycopersicum* can be a smart strategy to mitigate harmful impact of LT stress on detox system and overall growth of plant.

## Background

Different environmental factors end up being the source of plant stress, which has a negative influence on their metabolism, growth and even results in death [1]. Salinity, heavy metals, temperature, drought, and other abiotic factors are major factors that affect crop productivity [2]. Plants possess system that allow them to modify their physiology and growth in order to adapt to various environmental situations. Abiotic stressors, such as those brought on by salt, temperature, heavy metals, drought, etc., pose threat to plants because they harm their cells and prevent them from performing their regular physiological functions which reduces their output [3–5]. Low temperature exposure causes oxidative damage to plants. The antioxidant machinery of plants is turned on to restore the plants natural functioning. Antioxidants are essential for cellular redox equilibrium maintenance, low temperature (LT) stress tolerance, and cold adaptation [6]. Various metabolic and physiological activities in plants are connected to phytohormones [7]. By responding to the signaling cascades in plants, phytohormones have vital role in triggering the multiple intricate progression of development, growth and reaction to stressor. Additionally, these phytohormones can reduce the negative consequences of abiotic stress [8]. Abiotic stress signaling is mediated by plant growth hormones like jasmonic acid (JA), gibberellin, auxin, cytokinin, ethylene, salicylic acid (SA), abscisic acid (ABA) and brassinosteroid [9–11]. Under unfavorable climatic conditions, the capacity to endure abiotic stress is significantly mediated by plant hormones [12]. Amongst all phyto-hormones, Jasmonates has seen to have considerable importance. Jasmonates are ubiquitous, signaling molecules present in plants. It is mainly associated with abiotic stress response including triggering of antioxidant system of plants, stomatal movements, sugar and amino acid formation [13]. JA also interacts with different transcription factors and other phytohormones [14]. JA is having important role under stressful and normal conditions [15]. Stress in plants is brought on by extreme temperature differences. Plants normal function is altered by temperature stress (cold/low) [16]. Therefore, JA plays a part in reducing the impact of severe temperatures on plants [17]. Methyl jasmonate (MeJA), despite other jasmonates are obtained from fungi, is extricated from the jasmine (*Jasminum grandiflorum*) petals [18]. Flowers and reproductive parts often contain it, but mature leaves and roots retain it in trace amounts. JA can either increase or decrease response of plant depending upon its production [10].

Tropical and subtropical plants lack cold-mitigation mechanisms and are inherently vulnerable to cold. Whereas vegetation in temperate regions can typically endure cold stress. Tomato, soyabean, rice, potato, cotton and corn are among the crops that are chilling sensitive plants [19]. But like many other crops tomato is also cold-sensitive that exists among crops that are unable to withstand against LT stress [19]. Depending on cultivar, the temperature for development of fruit is often between 15–25 °C. There many problems associated fruit formation at low temperatures that includes anther dehiscence, viability of pollens etc. Among different cultivars of *S. lycopersicum* most of them are prone to LT stress during all developmental stages including vegetative and reproductive phase [20].

To determine the effect of foliar MeJA application in mediating LT stress in *S. lycopersicum*, a controlled experiment was conducted. Here, we postulate that varied MeJA treatment doses may result in the same response to LT stress by controlling growth, the stress response, and antioxidant capacity. The precise goals that were assessed were: 1.) Exogenous application of MeJA at various concentrations during LT stress; and 2.) Choosing the optimal concentration in accordance with probable adaptation processes including antioxidant potential, growth, and stress indicators equivalent to those of controls. This study offers the groundwork for the creation of preventative measures against LT stress in the pusa sheetal cv. of tomato and will aid in the framing of various omics-based LT stress management techniques. The determination of this work was to unravel the impact of different concentrations of MeJA on low temperature stressed *Solanum lycopersicum* and its potential role in mediating cold stress tolerance.

The aim of this work was to unravel the impact of different concentrations of MeJA on low temperature stressed *Solanum lycopersicum* and its potential role in mediating cold stress tolerance.

## Results

### Hydrogen peroxide (H_2_O_2_) level

**Fig. 1:**
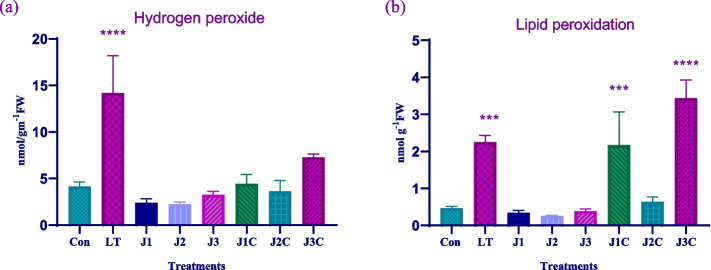
Influence of MeJA and LT stress on oxidative stress parameters: For the experiment, seedlings older than forty days were used. After three days of restoration determination of **a** hydrogen peroxide content **b** lipid peroxidation was carried. The statistics shows the mean and standard deviation of three replicates using significance level (*p* < 0.05). Data with an asterisk (*) as determined by Dunnet’s multiple comparison. Control: 25/18 °C + 0 µM MeJA, LT: 10/3 °C + 0 µM MeJA, J1: 25/18 °C + 5 µM MeJA, J2: 25/18 °C + 10 µM MeJA, J3: 25/18 °C + 15 µM MeJA, J1C:10/3 °C + 5 µM MeJA, J2C: 10/3 °C + 10 µM MeJA, J3C: 10/3 °C + 15 µM MeJA

ROS are produced when LT stress occurs, and this raises the quantity of H_2_O_2_. In our current study we observed that H_2_O_2_ levels change in response to LT stress and MeJA supplementation. H_2_O_2_ content increased in LT only plants as that of control plants by 3.4 fold. However, decrease in H_2_O_2_ content was seen in J1, J2, and J3 treated plants by 0.5 fold, 0.5 fold and 0.2 fold respectively. As that of LT and MeJA treated plants J1C, J2C and J3C showed 3.2 fold, 3.9 fold and 1.9 fold reduction in H_2_O_2_ content (Fig. 1a). Extreme decrease was seen among J2C plants. Thus, we come to the conclusion that MeJA helps to reduce the H_2_O_2_ buildup in *S. lycopersicum.*

### Lipid peroxidation level

MDA concentration indicates that LT stress is related with cellular membrane damage. The development of TBARS is used to determine MDA levels. Amount of TBARS in cold stressed plants was 5.3 fold more as that of control plant (Fig. 1b). In MeJA treated plants i.e. J1, J2 and J3 level of TBAR was declined by 0.27 fold, 0.47 fold and 0.20 fold respectively as that of control. However, MeJA and cold stressed plants i.e. J1C, J2C and J3C showed different response pattern. J1C treatment showed almost same level of peroxidation level as that of LT only plants. However, J2C observed a significant drop in TBAR content by 3.5 fold as that of LT plants. J3C treated plants have 0.65 fold more TBAR content as that of LT only plants. The J2C treatment produced the lowest TBAR content throughout all LT plants, whereas J3C plants had the highest levels. Plants that had been given MeJA were able to reduce the injury that LT stress caused to their membranes.

### Glutathione reductase (GR) activity

**Fig. 2:**
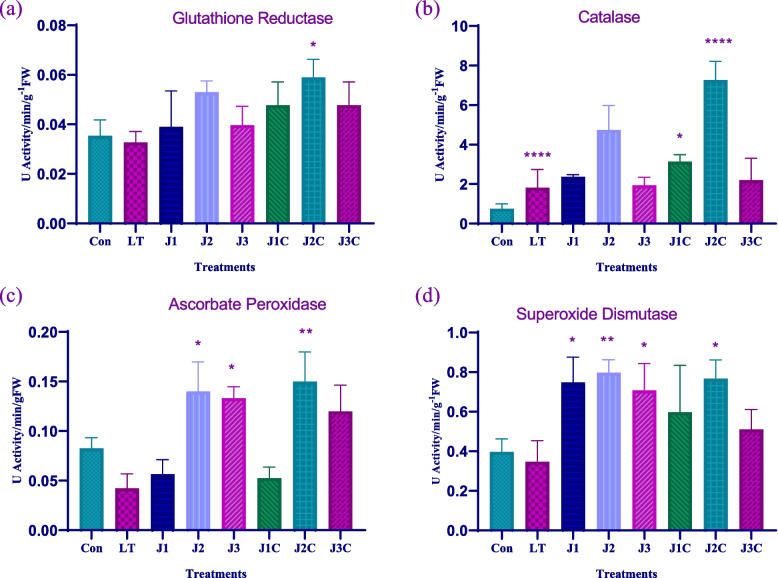
Effect of LT stress and MeJA on antioxidant enzyme activity: For the experiment, seedlings older than forty days were used. After three days of restoration determination of **a** GR **b** CAT **c** APX **d **SOD was carried. The statistics shows the mean and standard deviation of three replicates using significance level (*p* < 0.05). Data with an asterisk (*) as determined by Dunnet’s multiple comparison. Control: 25/18 °C + 0 µM MeJA, LT: 10/3 °C + 0 µM MeJA, J1: 25/18 °C + 5 µM MeJA, J2: 25/18 °C + 10 µM MeJA, J3: 25/18 °C + 15 µM MeJA, J1C:10/3 °C + 5 µM MeJA, J2C: 10/3 °C + 10 µM MeJA, J3C: 10/3 °C + 15 µM MeJA

The enzyme GR is involved in the conversion of glutathione (GSSG) from its oxidized state to its reduced form (GSH). As a result, it keeps the pool of GSH/GSSG high, which is essential for developing tolerance to LT stress. Control plants show 0.1 fold increase in level of GR activity as that of LT plants. However, in plants treated with GSH, it raised by 1.1, 1.5, and 1.1 folds, respectively in comparison to control plants. When compared to LT only, GR activity enhanced in LT + MeJA treated plants including J1C, J2C, and J3C by 1.5 fold, 1.8 fold and 1.4 fold, respectively. J2C has the most GR activity, whereas LT only has the least (Fig. 2a). Thus, exogenous treatment of MeJA boosted GR activity in plants.

### Catalase (CAT) activity

Both LT stress and the MeJA treatment had an impact on the CAT enzyme activity in the leaves of seedlings of *S. lycopersicum*. Treating LT and non LT plants with MeJA resulted in increase in CAT activity. MeJA treated plants J1, J2, and J3 showed 3.0 fold, 6.2 fold and 2.5 fold increase in CAT activity, respectively, when compared with the control plants. Subtle increase in CAT activity (0.4 fold) as compared to control plant was also seen in LT only (Fig. 2b). Meanwhile, in LT + MeJA supplemented plants 1.7 fold, 4.0 fold and 1.2 fold rise in CAT activity was attained respectively. J2 concentration boosts the CAT activity under both standard and LT stress conditions. As a consequence, J2 concentrations significantly boosted CAT activity under both normal and LT stress conditions.

### Ascorbate peroxidase (APX) activity

H_2_O_2_ builds up in the plants as a result of extended LT stress. The APX enzyme is the scavenger of H_2_O_2_. MeJA treatment of plants improved the activity of APX. J1, J2 and J3 showed 0.68 fold, 1.7 fold and 1.5 an elevation in APX activity in comparison to the control. LT shows 0.5 fold decrease in APX activity. But LT + MeJA that includes J1C, J2C and J3C plants show 1.2 fold, 3.5 fold and 2.8 fold increase respectively compared to that of LT only. Thus, J2C have the most AXP activity (Fig. 2c). As a result, LT stress causes a decrease in APX activity, whereas JA treatment boosts it in both LT and normal conditions.

### Superoxide dismutase (SOD) activity

SOD is reported to be key player of defense in plant system against superoxide free radicals produced by LT stress is assumed to be SOD enzyme. MeJA treatment resulted in enhanced SOD activity in plants relative to control plants, whereas LT plants exhibited decreased SOD activity. Only the MeJA-treated plants J1, J2, and J3 demonstrated 1.7, 2.0, and 1.7 fold increases in comparison to the control, respectively. LT + MeJA treated plants, including J1C, J2C, and J3C, show SOD activity that is 1.5 fold, 2.10 fold, and 1.7 fold than that of LT only plants (Fig. 2d). J2C plants had the highest overall SOD activity, whereas LT plants had the lowest. MeJA caused an increase in SOD activity.

### Growth aspects

**Fig. 3:**
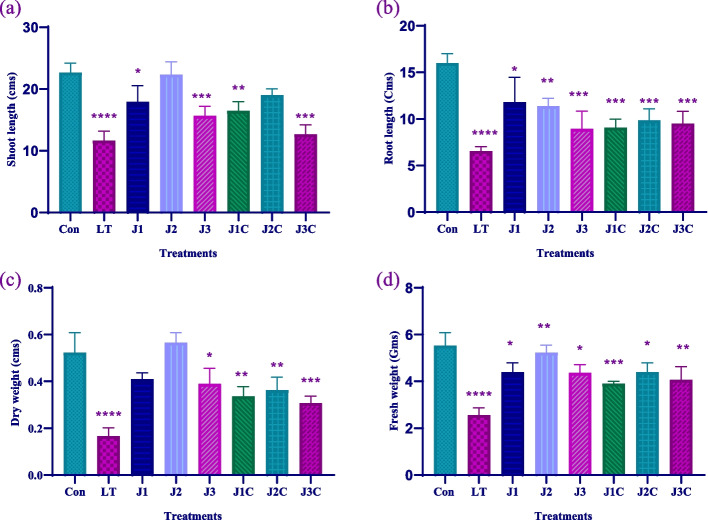
Effect of LT stress and MeJA on growth aspects: For the experiment, seedlings older than forty days were used. After three days of restoration determination of growth parameters including **a** Shoot length **b** Root length **c** Dry weight and **d** Fresh weight was done. The statistics shows the mean and standard deviation of three replicates using significance level (*p* < 0.05). Data with an asterisk (*) as determined by Dunnet’s multiple comparison test. Control: 25/18 °C + 0 µM MeJA, LT: 10/3 °C + 0 µM MeJA, J1: 25/18 °C + 5 µM MeJA, J2: 25/18 °C + 10 µM MeJA, J3: 25/18 °C + 15 µM MeJA, J1C:10/3 °C + 5 µM MeJA, J2C: 10/3 °C + 10 µM MeJA, J3C: 10/3 °C + 15 µM MeJA

**Fig. 4:**
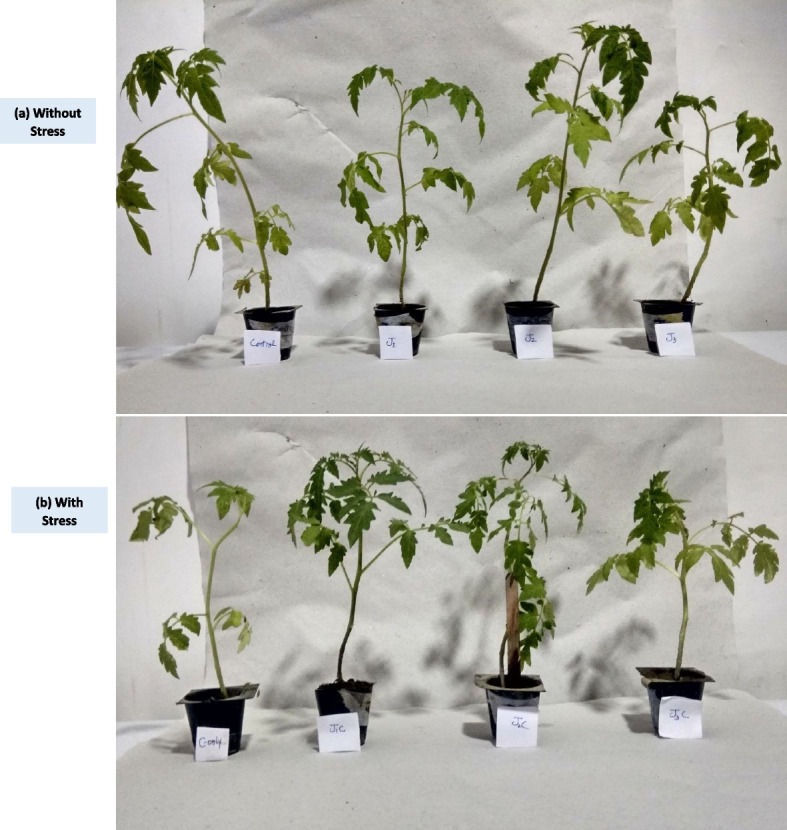
Effect of varying MeJA concentrations and cold stress on shoot morphology. Morphology of shoot of Forty days old seedling **a** without stressed and **b** Post 3 days LT stress recovery Control (Con): 25/18 °C + 0 µM MeJA, J1: 25/18 °C + 5 µM MeJA, J2: 25/18 °C + 10 µM MeJA, J3: 25/18 °C + 15 µM MeJA, Low temperature stress (LT/C): 10/3 °C + 0 μM MeJA J1C: 10/3 °C + 5 µM MeJA, J2C: 10/3 °C + 10 µM MeJA, J3C: 10/3 °C + 15 µM MeJA

**Fig. 5:**
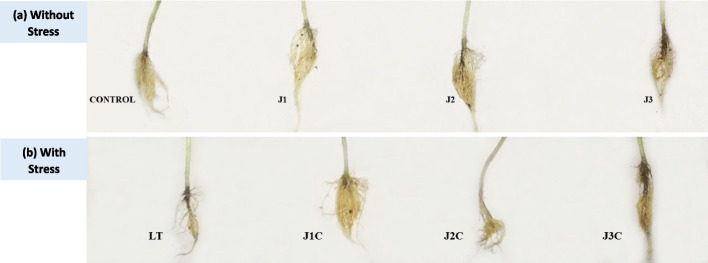
Changes in root morphology caused by LT stress and MeJA concentrations. Root Morphology of shoot of Forty days old seedling **a** under non stressed and **b** Post 3 days stress recovery. Control (Con): 25/18 °C + 0 μM MeJA, Low temperature stress (LT): 10/3 °C + 0 μM MeJA, J1: 25/18 °C + 5 µM MeJA, J2: 25/18 °C + 10 µM MeJA, J3: 25/18 °C + 15 µM MeJA, J1C: 10/3 °C + 5 µM MeJA, J2C: 10/3 °C + 10 µM MeJA, J3C: 10/3 °C + 15 µM MeJA

In comparison to treated and control plants, LT plants demonstrated notable reductions in almost all growth indicators affecting total fresh and dry mass, root and shoot length, and plant size. Root length decreased in all aspects as compared to that of control. However, J1, J2 and J3 plants show 0.73 fold, 0.70 fold and 0.55 fold drop respectively in comparison to control plants. However, control plants shows 2.4 fold increase as compared to LT only. J1C, J2C and J3C plants showed 1.3 fold, 1.5 fold and 1.4 fold rise in root length when compared to LT plants (Figs. 3a and 4). The shoot length of MeJA treated plants except J2 was decreased by 0.79 fold, and 0.96 fold in J1 and J3, respectively as compared to control. On contrary, J2 plants showed significant surge by 0.9 fold as compared to control plants. J1C, J2C and J3C plants displayed 1.4 fold, 1.6 fold and onefold increase as that of LT plants respectively (Figs. 3b and 5). Fresh weight of plants was less as that of control except J2. J1 and J2 shows 0.8 fold and 0.7 fold decrease in FW. While as compared to LT only plants control, J1C, J2C and J3C shows 2.2 fold, 1.5 fold, 1.7 fold and 1.6 fold increase in FW respectively (Fig. 3d). While dry weight increased as compared to control by onefold in J2 treatment. But decreased by 0.77 fold and 0.75 fold under J1 and J3 treatment respectively. LT stressed plants treated with MeJA exhibit a considerable decrease in DW. However, J1C, J2C and J3C plants show 2 fold, 2.2 fold and 1.8 fold increase in DW as that of LT only (Fig. 3c).

### Stress tolerance index

**Fig. 6:**
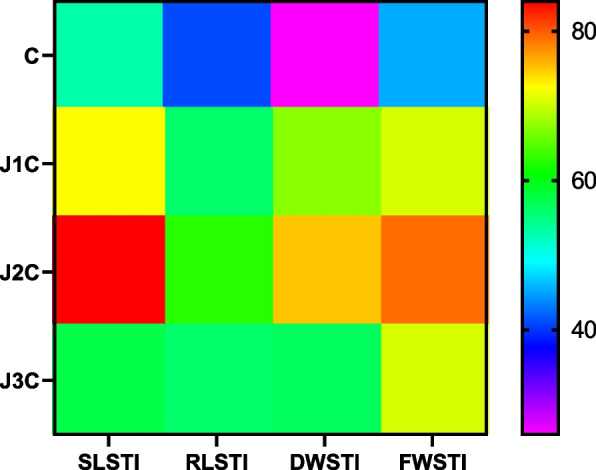
Heat map of STI: Based on several indicators of tolerance against stress (SLSTI, RLSTI, DWSTI, and FWSTI), the heat map of STI was developed, with blue denoting the least and red representing the most capacity for tolerance. Low-temperature stress (LT): 10/3 °C + 0 µM MeJA, J1C:10/3 °C + 5 µM MeJA, J2C: 10/3 °C + 10 µM MeJA, J3C: 10/3 °C + 15 µM MeJA

The *S. lycopersicum* (pusa sheetal cv.) has decreased tolerance threshold to cold stress. Exogenous MeJA supplementation, however, tends to raise its degree of tolerance. The highest values of the RLSTI and SLSTI are observed in J2C plants (61% and 79.5%, respectively), while the lowest values are observed in LT stress plants (41% and 52%, respectively). The maximum FW and DW values were found in J2C plants (79% and 69.5%, respectively). The LT stressed plants had the lowest values of FW and DW (46% and 33%, respectively). J1C plants displayed marginal RLSTI (56.5%), SLSTI (72.8%), FWSTI (70.5%), and DWSTI (64.5%). RLSTI (59.2%), SLSTI (75%), FWSTI (56%) and DWSTI (58.8%) were found in J3C plants (Fig. 6).

### Gas exchange parameters

**Fig. 7:**
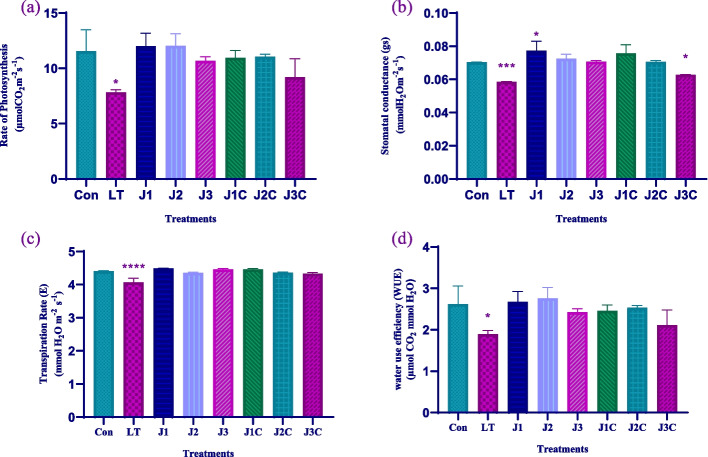
Effect of LT stress and MeJA on photosynthetic features: For the experiment, seedlings older than forty days were used. After three days of restoration determination of antioxidant activity of **a** Rate of photosynthesis **b** stomatal conductance **c** Rate of transpiration and **d** water use efficiency was done. The statistics shows the mean and standard deviation of three replicates using significance level (*p* < 0.05). Data with an asterisk (*) as determined by Dunnet’s multiple comparison. Control: 25/18 °C + 0 µM JA, LT: 10/3 °C + 0 µM MeJA, J1: 25/18 °C + 5 µM MeJA, J2: 25/18 °C + 10 µM MeJA, J3: 25/18 °C + 15 µM MeJA, J1C:10/3 °C + 5 µM MeJA, J2C: 10/3 °C + 10 µM MeJA, J3C: 10/3 °C + 15 µM MeJA

All of the gaseous exchange parameters that were assessed for all treatments showed notable variations. The net photosynthetic rate (PN) of LT plants was markedly lower (7.88 µmolCO_2_ m^−2^ s ^−1^) than control group of plants (11.56µmolCO_2_m-^2^ s^−1^). MeJA treated plants J1, J2 and J3 plants have 12.02µmolCO_2_m-^2^ s^−1^, 12.03µmolCO_2_m-^2^ s^−1^, and 10.6µmolCO_2_m-^2^ s^−1^ PN rate respectively. While LT + MeJA plants including J1C, J2C and J3C have 11.9µmolCO_2_m-^2^ s^−1^, 12.07µmolCO_2_m-^2^ s^−1^, and 9.22µmolCO_2_m-^2^ s^−1^, respectively. When compared to control plants, LT plants stomatal conductance (gs) was found to be considerably reduced (0.058mmolH_2_Om^−2^ s^−1^) than that of control plants (0.070 mmolH_2_Om^−2^ s^−1^). MeJA treated plants J1, J2 and J3 plants have 0.077 mmol H_2_O m^−2^ s ^−1^, 0.072mmolH_2_Om^−2^ s^−1^ and 0.070mmolH_2_Om^−2^ s^−1^, respectively. However, it was found that LT stressed plants exhibited lower transpiration rates (E) than control and LT + MeJA plants. J1, J3 plants have equal transpiration rate of 4.5 mmolH_2_Om^−2^ s^−1^. J2 have lesser rate of transpiration of 4.3 mmolH_2_Om^−2^ s^−1^. Water usage efficiency (WUE) was seen 2.6 µmolCO_2_m-^2^ s^−1^H_2_O in control plants. MeJA treated plants mostly J2 have 2.8µmolCO_2_m-^2^ s^−1^H_2_O while J1 and J3 have 2.6 µmolCO_2_m-^2^ s^−1^H_2_O and 2.4 µmolCO_2_m-^2^ s^−1^H_2_O, respectively. Moreover, J1C, J2C and J3C have 2.4 µmol CO_2_ mmol H_2_O, 2.6µmolCO_2_m-^2^ s^−1^H_2_O and 2.4µmolCO_2_m-^2^ s^−1^H_2_O and 2.1 µmolCO_2_m-^2^ s^−1^H_2_O correspondingly (Fig. 7a-d).

### 2D contour plotting based analysis

**Fig. 8:**
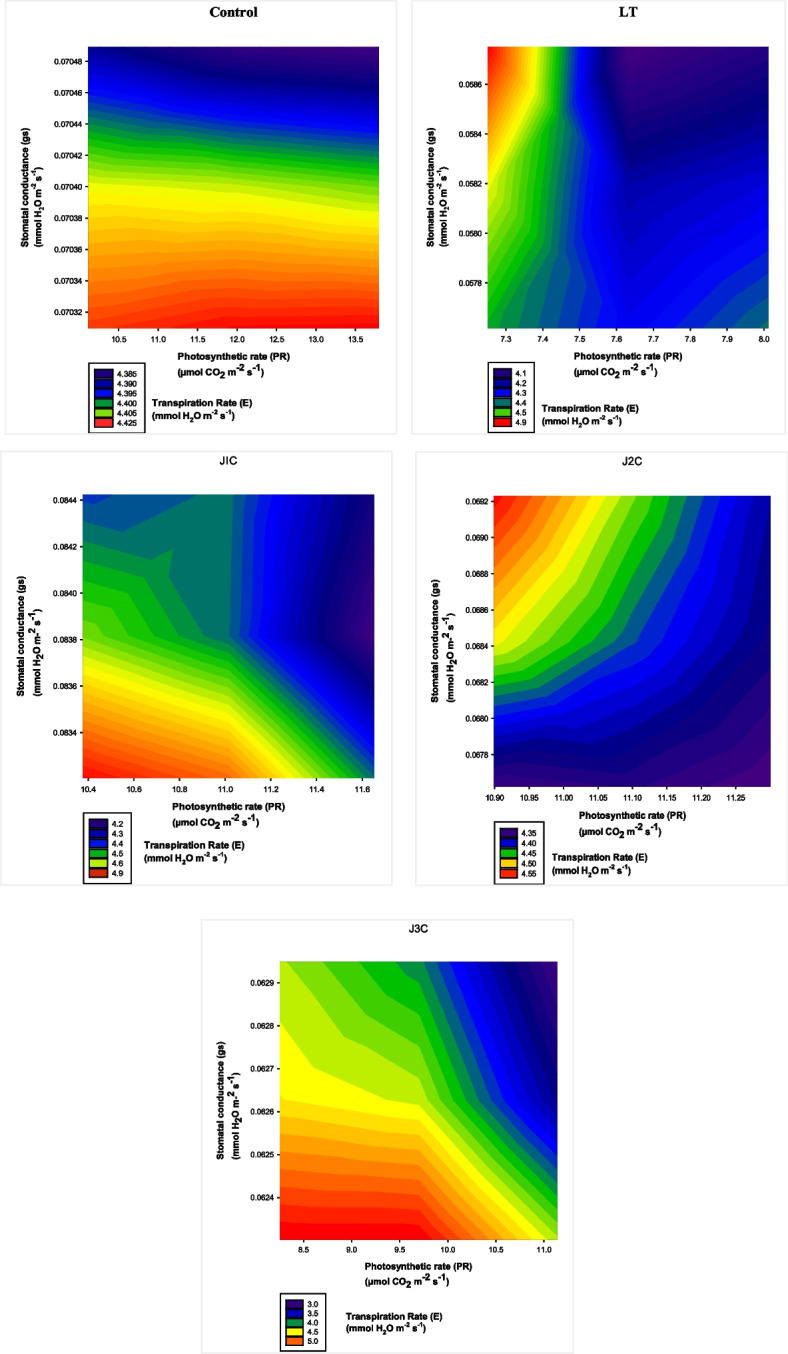
2d contour plots regarding interaction between stomatal conductance, transpiration rate and photosynthetic rate and in low LT stressed and distinct MeJA concentrations applied plants. Control (Con): 25/17 °C + 0 µM MeJA, LT: 10/3 °C + 0 µM MeJA, J1C: 10/3 °C + 5 µM MeJA, J2C: 10/3 °C + 10 µM MeJA, J3C: 10/3 °C + 15 µM MeJA

The response surface system is explained using 2D contour maps. Contrary to straight contour lines, which signify minor interactions, oval shapes indicate a significant interaction between the variables [21]. In this analysis, 2D pattern is used to visualize 3D form of data. The space between contour lines determines the sharpness in context to slope; the smaller the space, the steeper the slope (converting pattern of interplay), the more the space, the gentler the slope (lesser converting pattern), and the absence of contour lines denotes a flat area (consistent form of collaboration).The contour lines of control exhibit a standard pattern having a mild slope and flat sections at 4.4mmolH_2_Om^−2^ s^−1^and 4.3mmolH_2_Om^−2^ s^−1^ correspondingly. LT exclusively stressed plants, on the other hand, have a sharpness, steepness, and a noticeable departure from an oval form to a straight line in the contour lines. This indicates that transpiration rate, photosynthetic rate and stomatal conductance interact less. The outcome of J1C dose of MeJA on plant under LT stress display plots oval in shape initially. There is increase in steepness at 4.690 mmolH_2_Om^−2^ s^−1^ to 4.395mmolH_2_Om^−2^ s^−1^ with two minor flat regions. However, under J2C supplementation at transpiration rate 4.38mmolH_2_O m^−2^ s^−1^ displays flat zone, extremely oval shape and less steepness. This further supports the significant connection between transpiration rate, photosynthetic rate and stomatal conductance. J3C also shows a significant degree of steepness and an oval pattern deviation. Comparing this to J1C and J2C treatments, there is less interaction. As a result, in the present situation, J2C treatment produced the highest level of interaction, trailed by J1C and J3C, respectively, as that of LT stressed plant (Fig. 8).

## Discussion

Plant growth and development, as well as agricultural production, are severely hampered by the negative environmental effect of cold. The importance of MeJA in plant stress tolerance and adaptability is well known. JA may increase plant resilience to abiotic stressors. Plants go through significant physiological and metabolic changes in response to cold exposure to reduce oxidative damage [22]. Tomato is the one of the important crop due to its valuable composition but is susceptible to low temperature. LT stress triggers the production of ROS, which leads to an increase in H_2_O_2_ levels by several orders of magnitude. In our current study we observed that H_2_O_2_ levels change in response to LT stress and MeJA supplementation. These findings imply that MeJA could shield plants from cold stress by controlling the levels of H_2_O_2_ and TBARS. Consequently, the application of MeJA improves the cold tolerance of tomato plants by protecting them from oxidative damage.

According to recent reports, MeJA appears to boost plants antioxidant systems when they are under LT stress. Inducing the activity or gene expression of antioxidant enzymes, such as GR, CAT, SOD and APX is a critical component of plants defence mechanisms against oxidative stress. Improved membrane integrity and increased resilience to abiotic stressors, such as cold, were the results of the increased enzymatic antioxidant system [23]. In this study, all concentrations of MeJA applied topically increased antioxidant activity including GR, CAT, APX and SOD under both normal and LT stress conditions. Among different MeJA concentrations used, J2 concentration reduced oxidative stress much better than other concentrations. These findings correspond with those of Ma et al. [24], and Repkina et al. [25], those demonstrated that MeJA has a tendency to increase antioxidant activities in wheat under drought stress and cols stress respectively.

Plants have developed a variety of JA-mediated molecular biochemical and physiological processes to respond and adapt abiotic stresses in order to achieve tolerance to them. JA is a signaling molecule that promotes pathways for signal transduction in response to various abiotic stressors at low or high concentrations [26]. Exogenous JA treatment promotes leaf senescence via related the expression of genes in *Arabidopsis* plants that results in increased cold forbearance [27]. It was found that the low temperature had an impact on the growth on plant under study, MeJA treatment marginally increased the SL, RL, FW, and DW. In the current study, LT slowed seedling development and induced chilling damage, as evidenced by lower SL, RL, FW, and DW. The MeJA application, on the other hand, helped to minimize the negative consequences of stress [28]. However, MeJA decreased the growth of plants under normal conditions that is in accordance with previous studies [29].

One of the main physiological functions of plants is photosynthesis, which is dependent on a number of components including light, the fixation of CO_2_, and other abiotic factors including temperature [30]. According to previous studies, LT stress slows down the absorption of CO_2_, which lowers the rate of photosynthesis [31]. Compared to the control, LT stressed plants have the lowest rates of photosynthesis. Assessing the rate of photosynthesis under exogenously applied LT temperature stress with that under LT alone, MeJA tends to boost it. We also observed a considerable decrease in PN when tomato seedlings were cultivated at a low temperature. Stomatal conductance and transpiration rates were also significantly reduced under these circumstances. The plants therefore showed an increase in leaf WUE compared to LT stressed plants. So, the plants leaf WUE increased in comparison to their baseline values. Our findings also correlate with the MeJA decreased water loss in the leaves of wheat seedlings subjected to low temperatures by increasing WUE. This is accordance with Zahra et al. [32].

The complex process that allows the plant to survive in difficult conditions is the capacity of stomata to control their opening to reduce water loss by conserving CO_2_ absorption [33]*.* In response to changing environmental conditions, stomatal movements (opening and closing) regulate CO_2_ use and water loss through evaporation [34]. By restricting the intake of CO_2_, decreased stomatal conductance slows photosynthesis, whereas high stomatal conductance supports high photosynthesis production [35, 36]. The increased rate of CO_2_ absorption and subsequent increase in photosynthesis are caused by high stomatal conductance [34]. Stomata close because of the cold stress. Additionally, it has been noted that plants experience ABA accumulation while under LT stress. According to reports, the ABA improves stomatal closure. ABA also decreases GSH levels in guard cells. ABA levels can increase in response to JA treatment [37]. So, JA application under normal conditions with increasing concentration led to decline of stomatal conductance. But exogenous application of MeJA had promising effect under LT stressed conditions via increasing the stomatal conductance level as that LT stressed plants. Among different concentrations of MeJA used J2 have better results.

Straight lines indicate an unimportant kind of interaction, but the 2D contour maps reflect a substantial interaction between factors [21]. 2D contour plots were used to show the kind and degree of collaboration among stomatal conductance, photosynthesis and transpiration rate. According to contour plotting, these three characteristics interact significantly to reduce the capability of *S. lycopersicum* plants to survive under cold stress. In addition these results highlight the importance of the relationship between stomatal conductance, transpiration rate and photosynthetic rate in this case of J2C concentration. J3C also shows a significant degree of steepness and an oval pattern deviation. Comparing this to J1C and J2C treatments, there is less interaction. As a result, compared to stress plants that had received just LT treatment, J2C treatment produced the highest level of interaction in the present case, followed by J1C and J3C. The stress tolerance index (STI) is a helpful instrument for assessing the capacity for stress tolerance. Important characteristics for the categorization of tolerance are the RL, SL, DW and FW [38]. STI proposes a mechanism of tolerance that permits plants to continue to grow despite in the presence of abiotic stimuli like high metal stress [39]. Exogenously applied MeJA tend to surge the resistance level of *S. lycopersicum* under LT stress that is accordance with recent study [40]. J2C concentrations of MeJA under LT stress revealed the maximal level of stress tolerance. J2C concentrations of MeJA during LT stress showed the highest stress tolerance level.

## Conclusion

Exogenous MeJA treatment in the current study reduced the LT stress in the *Solanum lycopersicum* plant. The J2 concentration has a considerable chance of preventing the negative effects of LT stress. In contrast to other concentrations, the optimal concentration of MeJA has an array of potential roles in reducing LT stress, including maintaining membrane composition and osmotic balance (decreased H_2_O_2_ and TBARS), boosting antioxidant capacity (including GR, CAT, APX, and SOD), increasing growth characteristics (SL, RL, DW and FW), enhanced photosynthetic attributes, transpiration rate, stomatal conductance and WUE which leads to greater stress tolerance. Therefore, applying the appropriate amount of MeJA is crucial for assessing and improving stress tolerance in *Solanum lycopersicum*.

## Materials and methods

Seeds of the *Solanum lycopersicum* (Pusa Sheetal) that is cold susceptible were bought from Indian Agriculture Research institute (IARI), New Delhi, India and kept at seed repository in the Division of vegetable science, SKUAST- Kashmir. *Solanum lycopersicum* is cold susceptible plant that is mainly affected by low temperature dip at seedling stage. Seed surface decontamination with 2% sodium hypochlorite. These sterilized seeds after proper washing were sown in a growth chamber in a container with soil (pH 6.3) including peat and compost (4:1 V/V) and infused with sand (3:1 V/V). 40 days old seedling were used for experimentation. LT stress was applied to some plants for 24 h at 10^°^/3^°^C day/night temperature in a growth chamber, while the others were kept at 25/18 °C the standard temperature same as that of control. Three replicates of plants were maintained per group. However, foliar MeJA treatment to LT stressed (LT + MeJA) and non-stressed in preventive, preemptive and curative dosage-dependent manners of various concentrations of J1 (0.5 µM), J2 (10 µM), and J3 (15 µM). The MeJA supplemented LT stressed (LT + MeJA) and LT stressed (without MeJA) plants after 24 h of low temperature stress were maintained in the growth chamber at ambient day/night conditions with relative humidity of 75%, 700 molm^−2^ s^−1^ of photosynthetically active radiation, and standard day/night temperatures of 25/18 ± 3 °C. After three-day recovery period sampling was carried.

### Lipid peroxidation content

Amount of lipid peroxidation in plant leaf samples was conducted using Dhindsa et al., (1981) protocol. The samples were ground in a solution composed of thiobarbituric acid (TBA, 0.25%) in trichloroacetic acid (TCA, 10%). These samples were kept at 95ºC and cooled on ice followed by centrifuged at 10,000 g (10 min). The subsequent addition of 4 ml of a solution (20% TCA and 0.5% TBA) was added to 1 ml of supernatant. The non-specificity turbid was corrected by reducing the amount of absorbance value at 600 nm. The coefficient of extinction (155 mM^−1^ cm^−1^) was used to calculate TBAR content [38].

### Hydrogen peroxide (H_2_O_2_) content determination

The estimation of H_2_O_2_ content was evaluated by Okuda et al. Fresh sample of leaf tissue was grounded in chilled perchloric acid and centrifuged at 1300 g for 10 min. In order to neutralize the supernatant containing perchloric acid, 4 M potassium hydroxide was employed. By using centrifugation, the residual soluble perchlorate of potassium was eliminated. The final volume of about 1.5 ml have 3-methyl-2-benzothiazoline hydrazine (81 µl), phosphate buffer (0.375 M, Ph 6.5), extract eluate (1 ml), 3- (dimethylamino) benzoic acid (12.5 Mm, 400 µl) and peroxidase enzyme (0.25 U).The rise in value of absorbance was determined at 590 nm. The H_2_O_2_ amount was measured using standard calibration curve [39]_._

### Catalase (CAT)) assay

The Aebi (1984) procedure applied to assess the CAT enzyme activity. Samples of fresh leaves were crushed in extraction buffer containing 0.5 M Na-phosphate (pH 7.3), PVP (1% w/v), triton X 100 (1% v/v) and EDTA (3 mM). Next, centrifugation was carried out at 13280 g at 4 °C for 25 min [40]. The final volume consists of enzyme extract, H_2_O_2_ (3 mM), 0.5 M Na-phosphate buffer (pH 7.3) and EDTA (3 mM).The amount of activity of the CAT in the supernatant was determined by monitoring of the reduction in absorbance at 240 nm in correlation with the depletion of H_2_O_2_. For calculating purposes, the coefficient of absorbance was set at 0.036 mM^−1^.The proportion of enzyme needed to dispose off 1 µmol of H_2_O_2_ every minute deduces the enzyme in-terms of unit activity [40].

### Glutathione reductase (GR) assay

In order to determine glutathione reductase activity, 0.1 M potassium phosphate buffer (pH 7.2) with NADPH (0.2 mM) and oxidized glutathione (GSSG) (0.02 mM) were made. The reaction began after adding enzyme extract. The activity was determined through the measurement of the decline in absorbance at 340 nm for 3 min at 25 °C. The conversion of 1 µmol of GSSG min^−1^ results in one unit of enzyme activity [41].

### Superoxide Dismutase (SOD) assay

The method suggested by Dhinsa et al. was performed to assess the SOD activity. The complete reaction consists of sodium phosphate buffer (pH 7.5, 0.1 M), L-methionine (13 mM), PVP (1% w/v), extract of enzyme (0.1 ml) with equal volume of riboflavin (60 μM), NBT (2.25 mM), EDTA (3 mM), Na_2_CO_3_ (1 M) and 1.0 ml DDW. After that, samples were exposed to 15 W fluorescent lamp at 28 °C. At a wavelength of 560 nm, the absorbance of the non-irradiated samples and the absorbance of the irradiated samples were analyzed. Enzyme activity (1U) was defined by the amount of enzyme extract required to achieve a reduction (50% inhibition of colour) of NTB [38].

### Ascorbate peroxidase (APX) activity

Activity of APX was done in accordance with the Nakano and Asada protocol. Fresh leaf samples were centrifuged at 4 °C for 10 min at 7800 g in potassium-phosphate extraction buffer (0.1 M, pH 7.5), EDTA (3 mM), Triton X 100 (1%) and PVP (1%). The total reaction volume comprised of buffer (1 cm^3^), ascorbate (0.5 Mm), H_2_O_2_ (0.1 mM), EDTA (0.1 mM) and enzyme extract (0.05 cm^3^). For 5 min, the reaction was carried out at 25 °C. The APX activity in the supernatant was calculated using the decline in ascorbate absorbance at 290 nm. Using 2.8 mM^−1^ cm^−1^ coefficient of absorbance the APX activity was evaluated. One unit of enzyme equals the quantity needed to break down 1 µmol of ascorbate every minute [42].

### Growth aspects

#### Root length and shoot length

The root-shoot length defines the distance plant must grow from the tip of root to its highest growing point on the central axis. To prevent desiccation, plants were gently pull up, cleaned, and then kept on wet filter papers. The lengths of the roots and shoots were measured and recorded using a measuring scale in centimeters [43].

#### Dry and fresh weight

Plants were carefully uprooted, then properly washed to eliminate soil, and weighed. Balance was used to determine fresh weight. The plants were kept in hot air oven at 80 °C until a consistent weight was reached, their dry mass was determined [44].

#### Stress tolerance index

An important tool for assessing the level of tolerance under stressful situations is the stress tolerance index (STI) parameter. Tolerance level based on various growth factors were calculated using the procedure described by [35].

### Determination of gas exchange parameters

To determine gas exchange factors, fully expanded topmost plant leaves were evaluated using an infrared gas analyzer (IRGA, Model LI6400XT). The test was conducted between 11:00 and 12:00 at a light-saturating intensity, a leaf area of 2 cm^2^, a block temperature of 25 °C, a CO_2_ flow controller of 300 µmol per second, and a PAR of 1600 µmol photons per square centimeter per second. The IRGA was calibrated before the experiment began, which involved zeroing out, replacing drierite and soda lime. For recording leaf gaseous exchange properties such as stomatal conductance (gs) (mmol H_2_O m^−2^ s^−1^), photosynthetic rate (PN) (mol CO_2_ m^−2^ s^−1^), transpiration rates (E) (mmol H_2_O m^−2^ s^−1^), and water usage efficiency (WUE), the third healthy leaf from the apex was used (Relationship between photosynthesis and transpiration).

### Statistical analysis

Using the software tool Graph Pad Prism (version 8), data analysis was done and significant difference between parameters were calculated using ANOVA. Data here shows mean and standard deviation of three replicates. Data having (*) shows significance level as given by Dunnet’s multiple comparison. Contour plotting was done via sigma 14.5 software.

## Data Availability

The datasets used in the current study were not made publicly accessible as it is to be shared as Meta data sharing might help to promote the long-term goal of this study. However, the corresponding author could provide the datasets upon reasonable request.

## Abbreviations

JA: Jasmonic acid
MeJA: Methyl jasmonate
LT: Low temperature
SA: Salicylic acid
ABA: Abscisic acid
TBA: Thiobarbituric acid
TCA: Trichloroacetic acid
GSSG: Oxidized glutathione
STI: Stress tolerance index
H_2_O_2_: Hydrogen peroxide
SOD: Superoxide Dismutase
GR: Glutathione reductase
CAT: Catalase
APX: Ascorbate peroxidase
IRGA: Infrared gas analyzer
